# Utilization of Flotation Wastewater for Metal Xanthate Gel Synthesis and Its Role in Polyaniline-Based Supercapacitor Electrode Fabrication

**DOI:** 10.3390/gels11060446

**Published:** 2025-06-10

**Authors:** Atanas Garbev, Elitsa Petkucheva, Galia Ivanova, Mariela Dimitrova, Antonia Stoyanova, Evelina Slavcheva

**Affiliations:** 1 “Acad. Evgeni Budevski” Institute of Electrochemistry and Energy Systems (IEES), Bulgarian Academy of Sciences (BAS), Acad. G. Bonchev Str., bl. 10, 1113 Sofia, Bulgaria; e.petkucheva@iees.bas.bg (E.P.); galia.ivanova@iees.bas.bg (G.I.); mariela.dimitrova@iees.bas.bg (M.D.); antonia.stoyanova@iees.bas.bg (A.S.); eslavcheva@iees.bas.bg (E.S.); 2 Center of Competence “HITMOBIL”, (CoC) Acad. G. Bonchev Str. 10, 1113 Sofia, Bulgaria

**Keywords:** gel, Xanthates, PANI, supercapacitors, composite materials, wastewater treatment, flotation

## Abstract

The aim of this study is to explore the feasibility of using flotation wastewater from copper–porphyry ore processing to synthesize a gel that serves as a precursor for a polymer nanocomposite used in supercapacitor electrode fabrication. These wastewaters—characterized by high acidity and elevated concentrations of metal cations (Cu, Ni, Zn, Fe), sulfates, and organic reagents such as xanthates, oil (20 g/t ore), flotation frother (methyl isobutyl carbinol), and pyrite depressant (CaO, 500–1000 g/t), along with residues from molybdenum flotation (sulfuric acid, sodium hydrosulfide, and kerosene)—are byproducts of copper–porphyry gold-bearing ore beneficiation. The reduction of Ni powder in the wastewater induces the degradation and formation of a gel that captures both residual metal ions and organic compounds—particularly xanthates—which play a crucial role in the subsequent steps. The resulting gel is incorporated during the oxidative polymerization of aniline, forming a nanocomposite with a polyaniline matrix and embedded xanthate-based compounds. An asymmetric supercapacitor was assembled using the synthesized material as the cathodic electrode. Electrochemical tests revealed remarkable capacitance and cycling stability, demonstrating the potential of this novel approach both for the valorization of industrial waste streams and for enhancing the performance of energy storage devices.

## 1. Introduction

The growing global demand for efficient and sustainable energy storage systems has driven significant interest in supercapacitors, which offer high power density, a long cycle life, and rapid charge–discharge capabilities [1,2]. Among the critical components of these devices, the nature and design of electrode materials play a pivotal role in determining overall performance. Recent advances have focused on developing hybrid and nanostructured electrodes that synergize electric double-layer capacitance with pseudocapacitance to improve both energy and power densities [3,4].

Polyaniline (PANI), a leading conductive polymer, has attracted considerable attention due to its high specific capacitance, excellent conductivity, and ease of synthesis [5,6]. However, limitations such as poor mechanical stability and a limited cycle life restrict its standalone application. These issues are typically addressed by forming composites with nanostructured materials, especially transition metal oxides like RuO_2_, which offers exceptional pseudocapacitive properties [7]. Due to the high cost of RuO_2_, more accessible alternatives such as MnO_2_ [8], NiO [9], Fe_2_O_3_ [10], CuO [11], and ZnO [12] have been explored. These metal oxides enhance the redox activity and electrochemical stability of PANI-based composites. For example, a PANI/MnO_2_ composite retained 86.3% of its capacitance after 1000 cycles—far exceeding that of pure PANI [13]. Synergistic improvements are also observed when incorporating carbon-based materials like graphite and carbon nanotubes, achieving energy densities up to 318 Wh kg^−1^ and power densities of 12.5 kW kg^−1^ [14].

Importantly, the total pseudocapacitance of such hybrids is not merely additive; interfacial effects can significantly alter electrochemical behavior. For instance, PANI/MoO_3_ composites show diminished redox peaks compared to MoO_3_ alone, suggesting complex interfacial interactions that merit deeper investigation [15].

Simultaneously, growing environmental concerns regarding industrial wastewater—particularly from mining and mineral processing—have highlighted the need for circular economy approaches that integrate resource recovery with pollution mitigation [16,17,18]. Among these wastes, flotation wastewater from copper and molybdenum ore processing is especially problematic and underutilized. This effluent contains a mixture of heavy metal ions (e.g., Cu^2+^, Ni^2+^, Fe^3+^, Zn^2+^), organosulfur compounds such as potassium ethyl xanthate (C_2_H_5_OCS_2_K), flotation agents, and petroleum-based reagents. Such complexity poses environmental risks due to toxicity and persistence in ecosystems.

Xanthates, a key component of flotation reagents, are of particular interest. While they are toxic and environmentally persistent, they also form stable, redox-active metal–organic complexes with transition metals [19]. Given that only around 50% of the estimated 372 million tons of xanthates produced annually are consumed during flotation processes, efficient and sustainable removal strategies are urgently needed [20]. Conventional approaches—including oxidative degradation [21,22,23,24,25], adsorption on activated carbons and zeolites [26,27,28,29,30,31], and emerging technologies like membrane separation, photocatalysis, and electrochemical oxidation [32,33,34,35,36,37]—offer partial solutions. However, many are energy-intensive, single-use, or generate secondary waste.

A promising alternative lies in the valorization of flotation wastewater into functional materials. While solid industrial wastes like red mud, fly ash, and mine tailings have been successfully utilized for supercapacitor electrodes [16,17,38], flotation wastewater remains largely untapped. Its unique combination of redox-active xanthates and metal ions offers chemical richness that is ideal for functional material synthesis.

Recent studies suggest that metal–xanthate complexes can serve as redox-active building blocks in electrochemical materials [38,39,40]. These insights form the basis of the present study, which demonstrates for the first time the direct transformation of flotation wastewater into a redox-active metal xanthate (MX) gel via spontaneous redox interaction with nickel powder. The resulting gel, comprising nickel hydroxides, xanthate–metal complexes, and silicates, is subsequently integrated into a PANI matrix via oxidative polymerization. The resulting PANI–MX nanocomposite is tested as a positive electrode in an asymmetric supercapacitor, paired with YP-50F activated carbon.

Asymmetric supercapacitors, which combine a pseudocapacitive electrode with a capacitive counterpart, are known to provide wider voltage windows and enhanced energy density compared to symmetric systems [41,42,43,44]. Our results affirm these advantages, showing high specific capacitance, excellent rate capability, and robust cycling stability. The PANI-MX/YP-50F device illustrates the viability of flotation wastewater as a sustainable feedstock for high-performance energy storage systems.

Comparative analyses further emphasize flotation wastewater’s advantages over other waste-derived materials. Unlike red mud or mine tailings, flotation wastewater contains electrochemically beneficial organics such as xanthates, which contribute to enhanced structural integrity and redox activity in electrode materials [38,39,40]. Furthermore, the scalable, low-waste synthesis method described here directly supports global sustainability goals, providing an integrated solution to environmental remediation and energy storage.

## 2. Results and Discussion

### 2.1. Physicochemical Data

#### 2.1.1. XRD Analysis of the Precursor Gel

**Figure 1:**
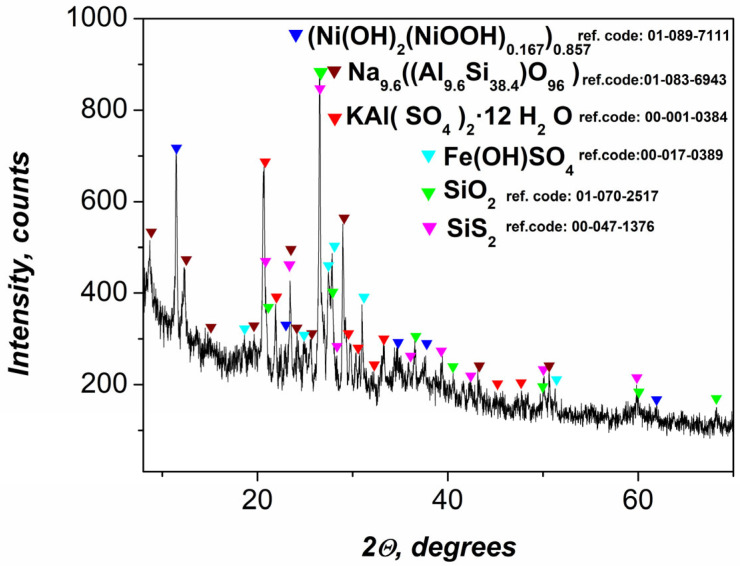
XRD data of prepared gel.

X-ray diffraction (XRD) analysis of the precursor gel (Figure 1) revealed the presence of multiple crystalline phases, reflecting its heterogeneous origin and complex chemical composition derived from flotation wastewater treatment. Among the most prominent diffraction peaks are those corresponding to the silicon-containing phases SiS_2_ and SiO_2_. While SiO_2_ is electrochemically inert, SiS_2_, being a semiconductor, may contribute marginally to the capacitive behavior—though its tendency to passivate under alkaline or neutral conditions limits this effect.

Well-defined peaks corresponding to a sodium aluminosilicate phase, identified as Na_9.6_Al_9.6_Si_38.4_O_96_, which is indicative of zeolitic or feldspathoid-type structures, as well as KAl(SO_4)2.12_H_2_O, are also observed. These mineral species may serve as mechanical supports and also contribute to ionic interactions within the matrix [44].

The active role of β-Ni(OH)_2_ and γ-NiOOH in the redox reactions that contribute to the electrochemical characteristics of composite materials is well known [45,46]. However, the diffractogram presented here does not show diffraction peaks corresponding to Ni(OH)_2_/NiOOH. Instead, a well-crystallized rhombohedral nickel-based phase, indexed as (Ni(OH)_2_(NiOOH)_0.167_)_0.857_, was identified. This phase exhibits distinct diffraction peaks at 11.43°, 22.98°, 34.57°, and 37.16° (2θ), corresponding to the (003), (006), (012), and (104) crystallographic planes, respectively. This specific composition has been associated with improved supercapacitor performance [47]. Despite the absence of distinct Ni(OH)_2_ and NiOOH phases, the observed pattern reflects an ordered, intergrown structure incorporating both Ni^2+^ and Ni^3+^ in a stoichiometric ratio consistent with partial oxidation. The high crystallinity of this phase explains both its electrochemical stability and its dominant contribution to the observed capacitance.

Another identified phase was iron hydroxysulfate (Fe(OH)SO_4_), which reflects the oxidation and precipitation products of Fe^3+^ ions in an acidic flotation environment. This compound may participate in further redox processes or influence charge transport properties when embedded in the polymer matrix [48]. The XRD results confirm that the gel precursor consists of both electrochemically active (Ni-based) and structurally supportive (silico-aluminate) phases, along with minor transition metal salts, making it a complex but functionally versatile component for nanocomposite fabrication. These findings further confirm the suitability of the material for supercapacitor electrodes, as the crystalline Ni(OH)_2_/NiOOH phases are well known for their high pseudocapacitive behavior, and the additional components improve mechanical integrity and electrochemical stability during cycling.

The morphology and elemental composition of the gel prior to polymerization were investigated using scanning electron microscopy (SEM) and energy-dispersive X-ray spectroscopy (EDS). EDS analysis confirmed the presence of a broad range of elements associated with both the original flotation wastewater and the introduced nickel precursor. The elemental composition of the precursor gel is summarized in Table 1.

The high oxygen content (41.12 wt%) and significant presence of nickel (21.34 wt%) confirm the formation of nickel hydroxides, likely Ni(OH)_2_, as a major inorganic component of the gel. The presence of sulfur and carbon also supports the incorporation of xanthate residues (C_2_H_5_OCS_2_^−^) from the flotation reagents, forming mixed organic–inorganic complexes. The elements Al, Si, and Ca likely originate from gangue minerals or residual processing agents. These results indicate that the precursor gel contains a structurally and chemically diverse composition, serving as a multifunctional component for further polymer matrix integration.

The structural features and compositional richness observed in the precursor suggest that, upon incorporation into the polyaniline matrix, the resulting nanocomposite is likely to exhibit synergistic effects in terms of conductivity, stability, and ion storage. Carbon (7.28 wt%) is attributed to residual organic flotation reagents, primarily xanthates, which are known to bind strongly with transition metals like Ni and Cu. The presence of sulfur (2.37 wt%) further supports the incorporation of xanthate-derived groups (–OCS_2_^−^), confirming their retention during the gel formation process. Significant quantities of aluminum (4.44 wt%) and silicon (11.98 wt%) point to the likely entrainment of aluminosilicate species, possibly from gangue minerals in the ore or process residues. Calcium (2.61 wt%), potassium (2.95 wt%), and sodium (2.39 wt%) may derive from the flotation depressants (e.g., CaO) and pH regulators used during ore beneficiation. Trace elements such as Fe (1.63 wt%), Mg (1.18 wt%), and Ti (0.22 wt%) are also present, indicating complex mineralogical diversity.

**Figure 2:**
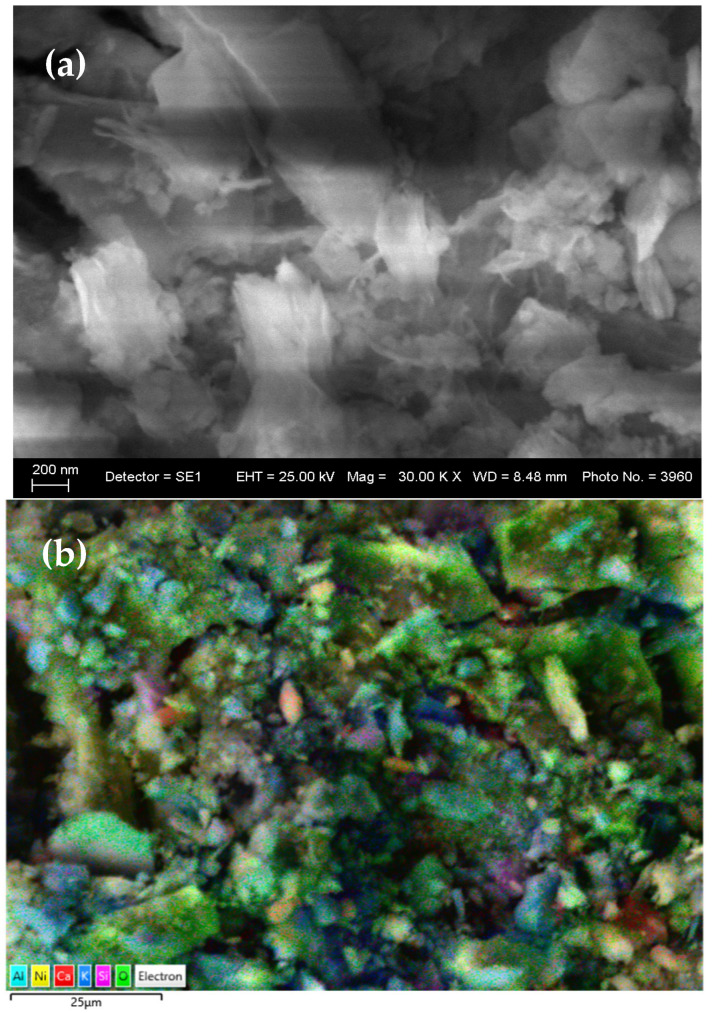
(**a**) SEM and (**b**) layered EDS images of the synthesized gel.

SEM images (Figure 2a,b) revealed a heterogeneous, porous structure, indicative of incomplete aggregation of inorganic phases and the presence of organic matrix residues. This morphology facilitates ionic mobility and suggests potential for enhanced electrochemical performance after polymerization.

#### 2.1.2. XRD Analysis of the Polymer Nanocomposite

**Figure 3:**
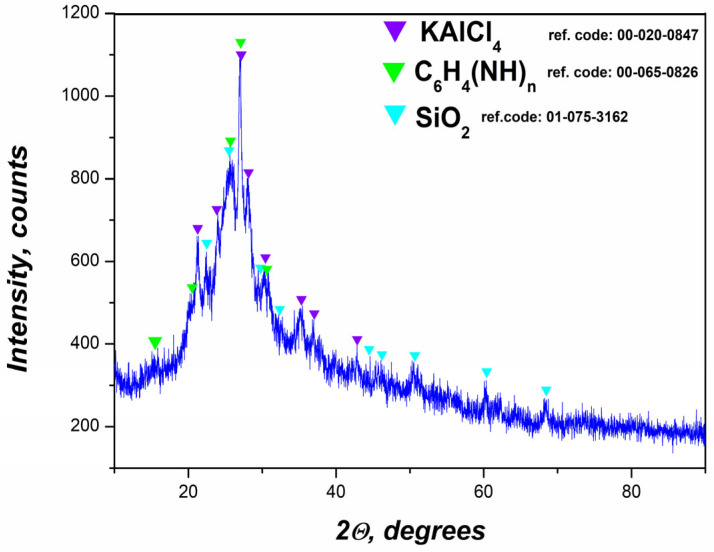
XRD data of prepared PANI-MX.

The X-ray diffraction pattern of the final nanocomposite, formed by embedding the flotation-derived gel into a polyaniline matrix, confirms the coexistence of both crystalline and amorphous components (Figure 3). Three main crystalline phases were identified—KAlCl_4_, C_6_H_4_(NH)_n_ (polyaniline), and SiO_2_—indicating a hybrid organic–inorganic structure with functional heterogeneity.

The presence of KAlCl_4_ suggests the retention or formation of aluminum–potassium–chloride complexes during the synthesis [49]. This phase results from ionic interactions between aluminum ions from the gel and chloride ions from the HCl medium used in the oxidative polymerization. These compounds act as dopants or stabilizers within the polymer matrix, potentially affecting conductivity and morphology.

The polymer matrix, represented by C_6_H_4_(NH)_n_, gives rise to broad diffraction features centered near 2θ ≈ 25°, typical of the emeraldine salt form of polyaniline. This broad halo is indicative of a semi-crystalline or amorphous polymer phase, in agreement with previous reports on conductive polyaniline systems [50].

The detection of SiO_2_ the form of amorphous or partially crystalline silica suggests the persistence of inorganic silicate species from the flotation process. The presence of silica can provide mechanical strength and thermal stability to the composite [51].

The elemental composition of the final polymer nanocomposite (PANI-MX), determined via energy-dispersive X-ray spectroscopy (EDS), is presented in Table 2. The analysis reveals a material dominated by carbon (82.96 wt%, 90.74 at%) and oxygen (5.95 wt%), consistent with the presence of a polyaniline matrix and oxidized species.

**Figure 4:**
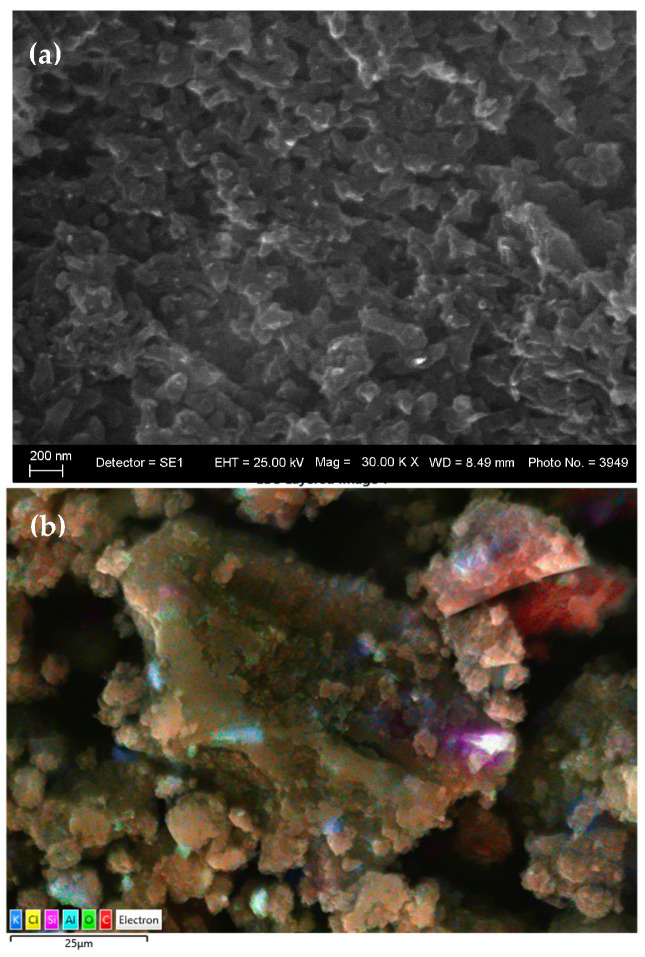
(**a**) SEM and (**b**) layered EDS images of the PANI-MX composite.

After the integration of the gel into the polyaniline matrix, EDS analysis shows a sharp increase in the carbon content (82.96 wt%), which corresponds to the formation of a polyaniline film on or around the gel particles Figure 4a,b). The significant decrease or disappearance of nickel and other metal elements from the surface spectrum (Ni: from 21.34 wt% to below the detection limit) suggests an effective encapsulation of the inorganic components in the polymer network. The appearance of chlorine (7.52 wt%) in the composition is related to the doping of polyaniline with Cl^−^ ions from the HCl medium, which is a known condition for obtaining the conductive “emeraldine salt” form. The presence of sulfur (S) and carbon fragments due to residual xanthate compounds (C_2_H_5_OCS_2_^−^) indicates their preservation in the gel phase and their participation in the final polymer structure. Xanthates probably form metal–organic complexes with Cu^2+^, Ni^2+^, and Fe^3+^ ions, which remain stable after polymerization and act as internal pseudocapacitive centers in the composite. These hybrid complexes contribute to the increase in overall capacitance through fast and reversible surface redox reactions, which builds on the basic charge storage mechanism characteristic of polyaniline.

Polymerization not only “seals” the gel in a conductive polymer shell but also preserves key functional groups from the waste environment, leading to the formation of a composite electrode with improved electrochemical activity and stability.

### 2.2. Electrochemical Results

Electrochemical evaluations, including cyclic voltammetry (CV), galvanostatic charge–discharge (GCD) measurements at 20 °C, and long-term cycling tests, were conducted on assembled asymmetric supercapacitor cells incorporating the synthesized material as the negative electrode. For comparison, symmetric supercapacitor cells using YP-50F activated carbon electrodes were tested under identical GCD conditions.

**Figure 5:**
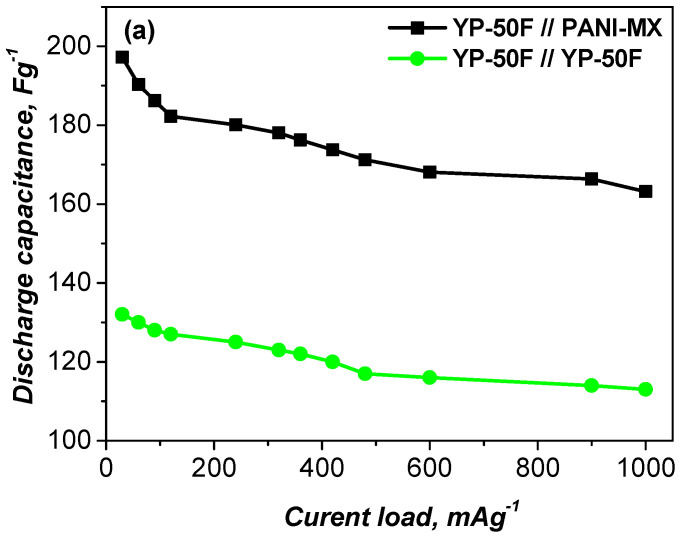

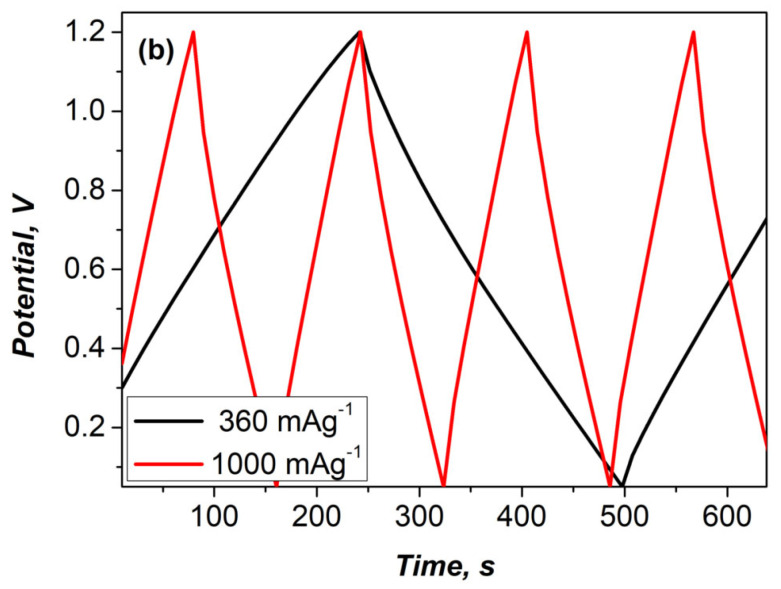
(**a**) Specific discharge capacitance as a function of current load for asymmetric and symmetric supercapacitors. (**b**) Voltage profiles of the asymmetric supercapacitor at 360 and 1000 mA g^−1^.

The results of the GCD measurements are shown in Figure 5a, illustrating the specific discharge capacitance as a function of current load in the range of 60 to 1000 mA g^−1^. Among the configurations tested, the asymmetric YP-50F//PANI–metal xanthate cell showed higher performance. In this configuration, the polyaniline–metal xanthate composite enhances the pseudocapacitance via synergistic Faraday redox reactions, while the activated carbon electrode contributes to the electrical double-layer capacitance due to its large surface area. This combination results in significantly improved electrochemical behavior. Furthermore, the presence of multiple metal cations in the wastewater likely facilitates the formation of a variety of metal–xanthate complexes, which further improve redox kinetics, electrical conductivity and overall capacitance. The coexistence of Faraday and non-Faraday processes in the asymmetric configuration contributes to the observed synergistic enhancement in charge storage efficiency.

The voltage profile of the asymmetric device (Figure 5b) displays characteristic features of a hybrid system and confirms the presence of low internal resistance (iR_360_ = 0.102 V and iR_1000_ = 0.253 V). This can be attributed to the homogeneous composition of the electrode and the fast surface redox kinetics at the negative electrode.

**Figure 6:**
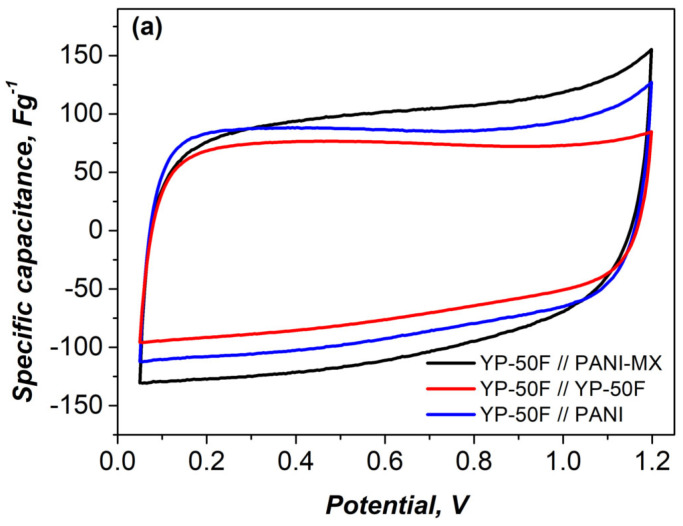

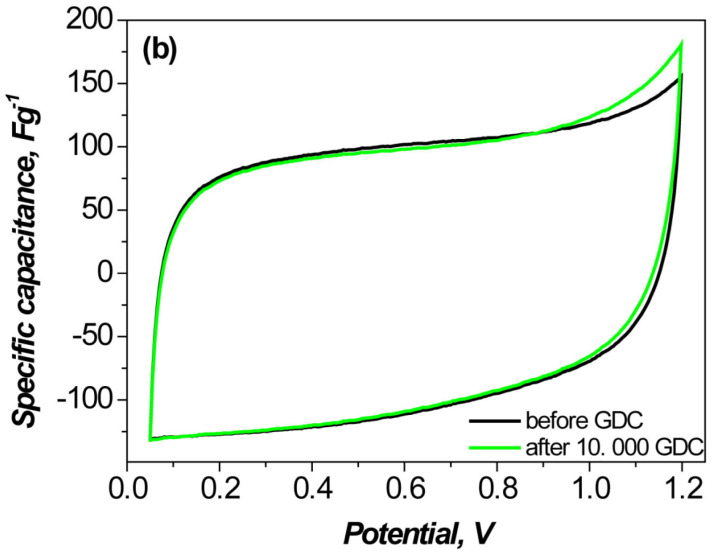
CV curves recorded at a scan rate of 10 mV·s^−1^: (**a**) symmetric and asymmetric supercapacitors before cycling; (**b**) asymmetric supercapacitor based on PANI–metal xanthate before and after 10,000 charge–discharge cycles.

The cyclic voltammetry (CV) profiles of the asymmetric YP-50F//PANI-MX cell remain nearly rectangular even at scan rates as low as 10 mVs^−1^ (Figure 6a), indicating excellent capacitive behavior and efficient charge storage kinetics [52]. In contrast, symmetric supercapacitor cells, although demonstrating good reproducibility of discharge capacitance values, exhibit limited overall charge storage capacitance, likely due to limited ionic diffusion and the lack of additional faradaic processes [53].

Furthermore, the asymmetric configuration using only PANI as the negative electrode exhibited a noticeably lower capacitance compared to the PANI-MX based cell, highlighting the beneficial role of incorporating metal xanthate in improving the electrochemical performance (Figure 6a).

**Figure 7:**
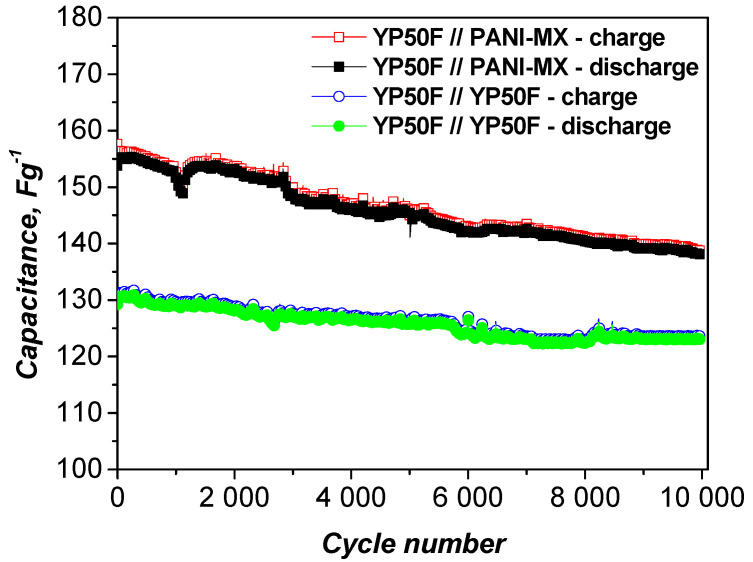
Long-term test of asymmetric and symmetric supercapacitor at 1000 mAg^−1^.

Figure 7 shows the capacitance retention of the asymmetric supercapacitor assembled with a composite of polyaniline and nickel xanthate as the negative electrode and activated carbon as the positive electrode. The long-term cycle stability was evaluated at a high current load of 1000 mAg^−1^. As shown, the device exhibits excellent durability, retaining 90.1% of its original capacitance after 10,000 charge and discharge cycles, along with a high Coulombic efficiency of 98%. This indicates that the PANI–metal xanthate composite retains its structural integrity and stable interfacial contact with the activated carbon electrode during continuous operation [54]. The stable cyclability is also confirmed by the CV curves (Figure 6b), which show minimal changes after 10,000 cycles, confirming the stable electrochemical activity and efficient charge storage behavior.

**Figure 8:**
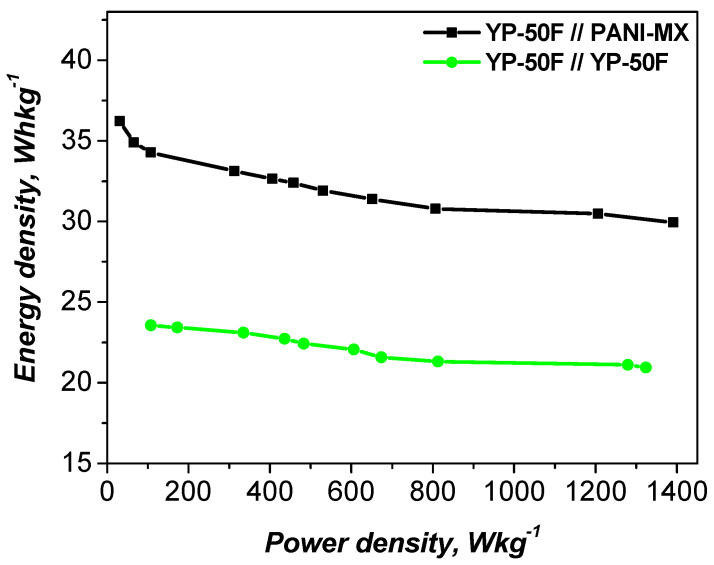
Ragone plot of asymmetric and symmetric supercapacitors.

The electrochemical performance of the studied materials was further evaluated using Ragone plots, with a comparison to the symmetric cell shown in Figure 8. The data indicate that the asymmetric configuration achieves a specific energy density of 35 Wh kg^−1^ at a power density of 100 W kg^−1^ and maintains a relatively high value of 32 Wh kg^−1^ even at 1200 W kg^−1^. In contrast, the YP-50F//YP-50F symmetric supercapacitor exhibits a lower energy density under the same conditions.

To facilitate comparison of the electrochemical performance, the key parameters have been summarized in Table 3.

Asymmetric and symmetric cells were evaluated in the same voltage range, which provided a direct and reliable comparison of their electrochemical characteristics. The improved energy and power performance of the asymmetric configuration was attributed to the synergistic interaction between the PANI–metal xanthate (PANI-MX) electrode, synthesized from flotation byproducts, and the YP-50F activated carbon electrode. In this configuration, the PANI-MX electrode undergoes Faraday redox reactions, contributing significant pseudocapacitance beyond the electrical double layer mechanism that dominates in symmetric cells. It is important to note here that the presence of multiple metal cations in the effluent allows the formation of diverse metal–xanthate complexes that enhance redox kinetics, conductivity, and overall capacitance. This compositional diversity offers a high degree of adaptability, improving the flexibility and overall efficiency of the asymmetric supercapacitor.

### 2.3. Comparative Assessment with Conventional Remediation Strategies

This study pioneers the direct utilization of flotation wastewater not merely as a contaminant-laden effluent but as a chemically active medium for synthesizing functional metal xanthate gel precursors. Unlike industrial wastes such as mining tailings or metallurgical sludge—which predominantly consist of inert minerals or oxidized particles—flotation wastewater is rich in redox-active species, including transition metal ions (Cu^2+^, Ni^2+^, Fe^3+^) and organosulfur compounds like potassium ethyl xanthate [55,56,57,58,59].

These xanthates, typically degraded or discarded during standard treatments, are preserved and repurposed here. Their ability to form stable, redox-active complexes contributes directly to the electrochemical performance of the final nanocomposite. In contrast, materials derived from tailings or sludge generally require energy- and reagent-intensive preprocessing (e.g., calcination, doping, acid leaching) to become electrochemically viable [60,61].

The gelation process developed in this study proceeds via spontaneous redox reactions between flotation wastewater constituents and sacrificial nickel powder, forming a nickel hydroxide/xanthate–metal matrix. This matrix is directly embedded into a conductive polyaniline (PANI) framework, enabling a dual-function approach: wastewater remediation and functional material synthesis—without the intermediate waste generation steps typical of conventional methods [62,63].

Benchmarking Against Conventional Remediation Techniques.

**(a)**  **Adsorption-Based Methods**

Widely used for heavy metal and organic contaminant removal, adsorption materials like activated carbon, MOFs, and engineered biochars act as passive scavengers. These are often discarded or require regeneration via energy-intensive means. Despite advances, such materials lack functional integration into energy devices [64,65].

In contrast, the metal xanthate gel developed here not only captures pollutants but is transformed into an active electrode material, effectively converting waste into a value-added nanocomposite.

**(b)**  **Precipitation and Coagulation**

Common in industrial treatment, chemical precipitation (e.g., with lime or sulfide) leads to metal-laden sludge, which is difficult to recycle and costly to dispose of [66]. Even electrocoagulation, while more efficient, generates solid waste and consumes significant energy.

The presented gelation method eliminates these drawbacks, forming no secondary sludge and converting waste ions into a stable electroactive network.

**(c)**  **Membrane-Based Technologies**

Technologies such as reverse osmosis and nanofiltration efficiently separate pollutants but suffer from membrane fouling, high operational costs, and waste brine generation [67,68]. These methods also lack material circularity.

Our approach is membrane-free, driven solely by spontaneous Ni^0^ oxidation, and yields electrochemically useful materials, bypassing all major membrane-related drawbacks.

**(d)**  **Circularity and Functional Integration**

Most remediation techniques aim solely to neutralize or remove pollutants. In contrast, this study’s gelation process operates within a circular economy framework, converting contaminants into useful functional components—notably, Ni(OH)_2_/NiOOH and metal xanthate redox centers within a PANI matrix. This functional integration is unique among remediation strategies and enhances both sustainability and material performance.

This aligns with the emerging literature on circular solutions in energy materials. For instance, Li et al. [69] developed Ni-doped carbon from mining wastewater for hybrid capacitors, Banerjee et al. [70] fabricated long-life PANI electrodes from industrial residues, and Liang et al. [71] demonstrated supercapacitors with superior cycling from waste-integrated polymers.

These studies validate the scalability and performance of waste-to-functional-material approaches, as exemplified by our method.

**(e)**  **Summary of Comparative Advantages**

Table 4 underscores the multifunctionality and environmental efficiency of our metal xanthate gelation strategy. This method offers not only pollutant removal but direct conversion into high-value energy materials, surpassing conventional approaches in both sustainability and utility.

## 3. Conclusions

This study demonstrates a sustainable and efficient approach for the utilization of flotation wastewater through the synthesis of a functional metal xanthate gel, which serves as a key component in high-performance polymer nanocomposite electrodes for supercapacitor applications. The gel, formed via the interaction of nickel powder with metal- and reagent-rich flotation effluents, incorporates nickel hydroxides, xanthate–metal complexes, and silicate residues into a structurally and chemically diverse matrix.

Upon integration into a polyaniline network via oxidative polymerization, the resulting nanocomposite exhibited a porous morphology, enhanced conductivity, and a stable crystalline–amorphous phase structure. Electrochemical characterization confirmed that the asymmetric supercapacitor configuration using this material achieved high specific capacitance, outstanding cycling stability (90.1% retention after 10,000 cycles), and superior energy and power densities compared to symmetric devices.

Importantly, the process also functions as a wastewater remediation pathway. Approximately 70–90% of the heavy metals (Cu, Mn, Pb, Fe) as well as part of the xanthate content are immobilized in the gel matrix, offering the dual benefit of pollution reduction and material recovery. This demonstrates high treatment efficiency with no need for high-temperature calcination or post-synthesis purification, reducing both energy input and operational cost.

The materials and procedures used—nickel powder, ambient-temperature gelation, and standard polymerization—are scalable and cost-effective. The process requires minimal energy, generates no secondary effluents, and aligns with industrial batch treatment systems. A preliminary economic analysis suggests that the cost of producing electrode-grade material from flotation wastewater is significantly lower than traditional methods involving metal oxide synthesis or carbon nanomaterial doping.

This work highlights a practical, circular economy strategy where a complex and hazardous industrial waste stream is converted into a value-added energy storage material. The proposed methodology addresses two global challenges simultaneously: sustainable energy storage and industrial wastewater pollution. It presents a scalable, environmentally beneficial, and economically feasible approach for large-scale adoption in both the energy and mining sectors.

## 4. Materials and Methods

### Materials and Reagents

**Figure 9:**
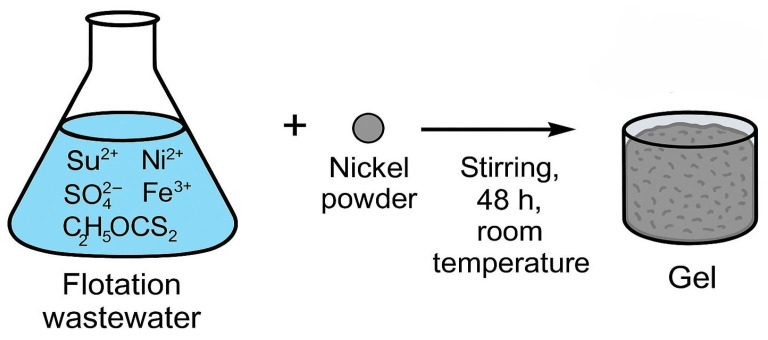
Mechanism of gel synthesis from flotation wastewater.

**Figure 10:**
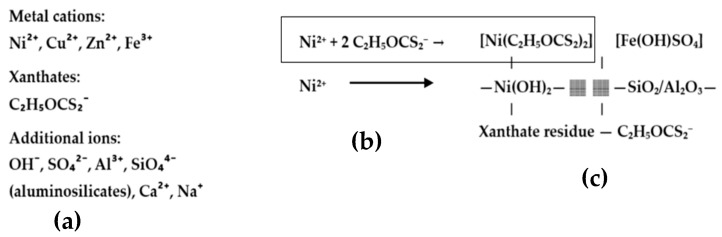
(**a**) Flotation water, (**b**) complexation of Ni^2+^ with xanthates, (**c**) MX gel = network of complex ions, hydroxides, minerals, and residual organic compounds.

The gel was synthesized by dispersing 0.07 g of nickel powder from Sigma-Aldrich^®^, St. Louis, MO, USA (M-Clarity™ quality level = MQ200) into 70 mL of flotation wastewater under continuous stirring at room temperature for 48 h. Following this process, the intermediate gel-like phase was allowed to settle and was then separated. The resulting 16 mL of gel contained nickel hydroxides, xanthate complexes, and embedded metal ions originating from wastewater. The mechanism of gel formation is shown in Figure 9. The gel matrix shown in Figure 10 is formed by coordination complexes such as [Ni(C_2_H_5_OCS_2_)_2_] and precipitated particles such as Ni(OH)_2_ and Fe(OH)SO_4_ embedded in a framework of amorphous silicates (SiO_2_/Al_2_O_3_). Ethyl xanthate residues (C_2_H_5_OCS_2_^−^) stabilize the gel network by coordinating with other metal cations and surface groups. This hybrid inorganic–organic structure provides both redox activity and mechanical stability, making it a multifunctional component for incorporation into conductive polymer matrices such as polyaniline.

Elemental analysis of the flotation wastewater was performed using Varian Vista MPX Simultaneous ICP-OES (Markham, ON, Canada) and ICP-MS (PlasmaQuant MS; Analytik Jena, Jena, Germany) to evaluate the retention efficiency of toxic and metal ions during gel formation. These data confirm the high retention capacity of the metal xanthate gel for a wide range of metallic and metalloid species, highlighting its potential for simultaneous environmental remediation and functional material synthesis. Table 5 shows the analysis of the water remaining in nature before and after gel synthesis.

**Figure 11:**
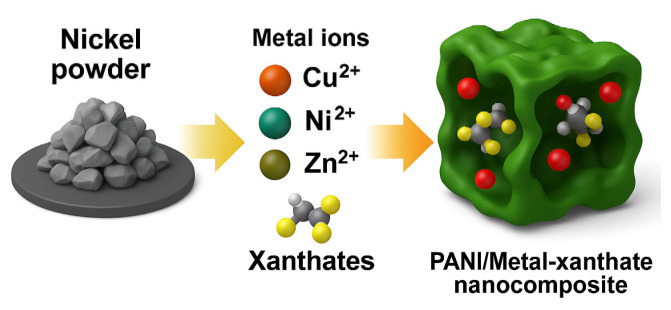
Graphical representation of the proposed structure of PANI-MX.

**Figure 12:**
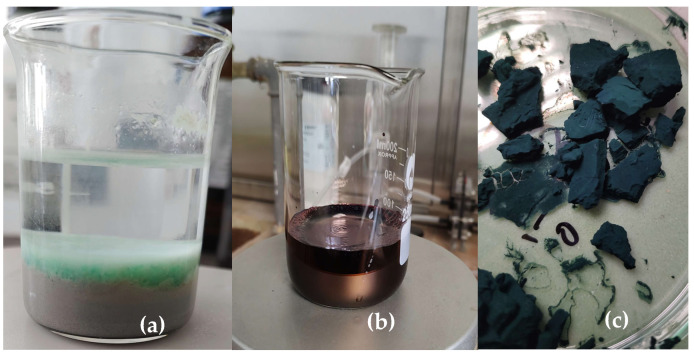
(**a**) The synthesized gel separating the two fractions of flotation wastewater, (**b**) oxidative polymerization of the MX gel, (**c**) PANI-MX.

**Figure 13:**
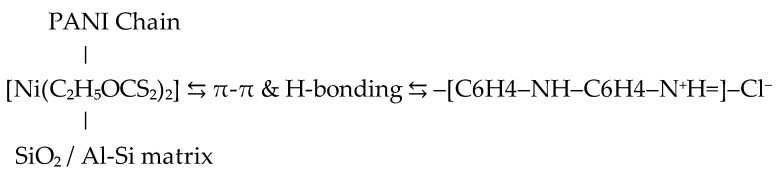
The gel is not only physically embedded but also chemically interacts with PANI through electrostatic bonding (between Cl^−^ and metal centers), π-π interactions between benzene nuclei, and possible hydrogen bonds between –OH and –NH.

The obtained gel was integrated into a polyaniline matrix Figure 11 via oxidative polymerization of aniline (0.2 M) using ammonium persulfate (0.25 M) as the oxidizing agent in 1 M hydrochloric acid (HCl), at ambient conditions in a ratio of 4 to 1. The resulting nanocomposite was dried in a vacuum dryer for 6 h at 65 degrees. (Figure 12). The nickel–xanthate complex [Ni(C_2_H_5_OCS_2_)_2_] in Figure 13 interacts with the growing polyaniline (PANI) chains through π–π stacking and hydrogen bonding, facilitating uniform encapsulation in the polymer. The presence of SiO_2_/Al-Si mineral residues from flotation wastewater enhances structural stability and provides additional surface anchoring. The resulting hybrid nanocomposite combines the redox activity of both components, improving charge transport and electrochemical performance. 

The resulting nanocomposite exhibited a compact, porous morphology and favorable adhesion characteristics (Figure 13).

The negative electrode was fabricated using the polyaniline electrode containing the synthesized gel derived from wastewater and polytetrafluoroethylene (PTFE) binder. The electrode mass loading was 30.93 mg cm^−2^. The positive electrode consisted of a composite made from YP-50F activated carbon (Kuraray Europe GmbH”, Main, Germany), acetylene black, and polytetrafluoroethylene (PTFE) binder. The active material was deposited on Ni foam as a current collector. After coating, both electrodes were dried under vacuum at 120 °C and pressed at 2 MPa to enhance mechanical stability.

**Figure 14:**
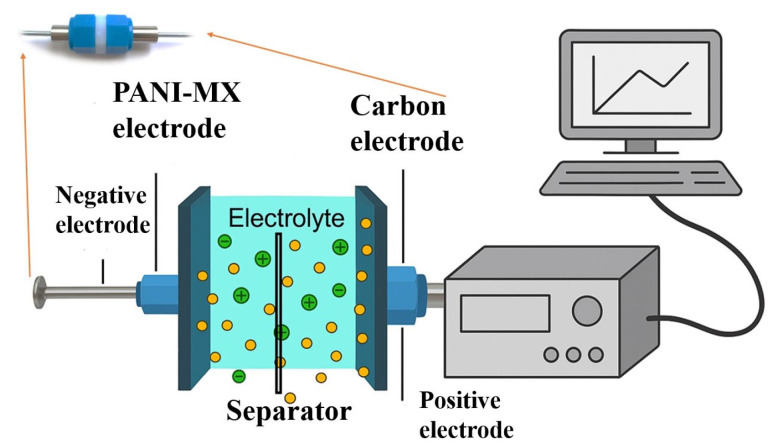
Schematic diagram of the experimental setup used for the electrochemical measurements.

The electrodes were assembled into a Swagelok-type two-electrode coin cell in an asymmetric configuration, using Viledon^®^ membrane as the separator and 6 M KOH, with a total volume of 0.003 mL applied during testing (Figure 14). For comparison purposes, symmetric cells consisting of two identical YP-50F-based electrodes were also prepared and evaluated under identical conditions. In addition, a cell was assembled with a negative electrode containing only polyaniline (PANI) in order to evaluate the individual contribution of the wastewater-derived gel to the overall performance.

The electrochemical measurements of the supercapacitors were conducted using cyclic voltammetry (CV) at a scan rate of 10 mV·s^−1^ in a voltage range of 0.05–1.2 V, both before and after long-term cycling. A Multi PalmSens system (Model 4, the Netherlands) was employed for the CV measurements. Galvanostatic charge–discharge (GCD) tests were performed using an Arbin Instrument System BT-2000 within the same voltage range (0.05–1.2 V). The GCD protocol included sequential current loads ranging from 60 to 1000 mA·g^−1^, with 30 cycles per step. For long-term cycling, the cells were subjected to 10,000 charge–discharge cycles at a constant current of 1000 mA·g^−1^.

The specific capacitance (Cs), derived from the cyclic voltammetry data, was calculated using the following equation, where I is the current, *dV/dt* is the voltage scan rate, and m is the mass of the active carbon material:

(1) $$C_{s}=\frac{4I\frac{dV}{dt}}{m}$$

The following equation was used to calculate the specific capacitance from the charge/discharge curves, where *I* (A), Δ*t* (s), *m* (g), and Δ*V* (V) indicate the discharge current, discharge time, mass of the active material, and voltage window, respectively:

(2) $$C\;=\;\frac{4I\Delta t}{mdv}$$

On the basis of the specific discharge capacitance, the energy density (E) and power density (P) are calculated using Equations (3) and (4):

(3) $$E\;=\;\frac{C\Delta V^{2}}{7.2}$$

(4) $$P\;=\;\frac{E}{t}$$

## Figures and Tables

**Table 1:** Map sum EDS spectrum of the synthesized gel.

Map Sum Spectrum					
**Element**	**Signal Type**	**Line**	**Wt%**	**Wt% Sigma**	**Atomic %**
C	EDS	K series	7.28	0.20	13.33
O	EDS	K series	41.12	0.12	56.54
Na	EDS	K series	2.39	0.03	2.29
Mg	EDS	K series	1.18	0.02	1.06
Al	EDS	K series	4.44	0.03	3.62
Si	EDS	K series	11.98	0.04	9.39
S	EDS	K series	2.37	0.02	1.63
Cl	EDS	K series	0.51	0.01	0.31
K	EDS	K series	2.95	0.02	1.66
Ca	EDS	K series	2.61	0.02	1.43
Ti	EDS	K series	0.22	0.01	0.10
Fe	EDS	K series	1.63	0.02	0.64
Ni	EDS	K series	21.34	0.07	8.00
Total			100.00		100.00

**Table 2:** Map sum EDS spectrum of the PANI-MX composite.

Map Sum Spectrum					
**Element**	**Signal Type**	**Line**	**Wt%**	**Wt% Sigma**	**Atomic %**
C	EDS	K series	82.96	0.10	90.74
O	EDS	K series	5.95	0.08	4.88
Na	EDS	K series	0.10	0.01	0.06
Mg	EDS	K series	0.10	0.01	0.05
Al	EDS	K series	0.57	0.01	0.28
Si	EDS	K series	2.17	0.01	1.02
Cl	EDS	K series	7.52	0.03	2.79
K	EDS	K series	0.34	0.01	0.11
Fe	EDS	K series	0.29	0.01	0.07
Total			100.00		100.00

**Table 3:** Summary of electrochemical performance parameters of the tested supercapacitor cells.

Supercapacitor	Specific Discharge Capacitance		Capacitance Retention, %		Energy Density	
	At 360 mAg^−1^	At 1000 mAg^−1^	After 1000 GCD	After 10,000 GCD	At 100 Wkg^−1^	At 1210 Wkg^−1^
Asymmetric	176.27	163.14	98%	90%	35.00	30.67
Symmetric	122.21	113.06	99%	95%	30.46	21.25

**Table 4:** Comparison of conventional remediation methods with the metal xanthate gelation approach.

Method	Typical Output	Reusability	Integration into Functional Devices	Energy/Resource Input	Waste Generation
**Adsorption**	Spent adsorbent	Low	Rarely	Moderate	High
**Precipitation**	Metal-rich sludge	None	No	High	High
**Membrane Separation**	Concentrate (brine/reject)	Limited	No	High	Moderate
**This Study**	Functional gel composite	High	**Yes—as electrode material**	Low	**Minimal**

**Table 5:** Elemental analysis of the flotation wastewater.

Element	Before Gel Synthesis mg/dm^3^	After mg/dm^3^
Aluminum (Al)	0.167	0.024
Arsenic (As)	0.009	0.007
Copper (Cu)	0.026	0.011
Iron (Fe)	0.043	0.010
Manganese (Mn)	0.012	0.003
Sulfur (S)	505.9	440
Lead (Pb)	0.003	0.001

## Data Availability

This article presents the original findings of the current study. The corresponding author can provide the data discussed in this publication upon request, as it is part of an ongoing investigation.

## Abbreviations

The following abbreviations are used in this manuscript:

PANI	Polyanyline
PANI-MX	PANI–metal xanthate composite
